# Supporting Pregnant Aboriginal and Torres Strait Islander Women to Quit Smoking: Views of Antenatal Care Providers and Pregnant Indigenous Women

**DOI:** 10.1007/s10995-013-1373-z

**Published:** 2013-10-23

**Authors:** Megan E. Passey, Rob W. Sanson-Fisher, Janelle M. Stirling

**Affiliations:** 1 University of Sydney, Lismore, NSW Australia; 2 University of Newcastle, Newcastle, NSW Australia

**Keywords:** Tobacco smoking, Smoking cessation, Indigenous, Consumer preference

## Abstract

To assess support for 12 potential smoking cessation strategies among pregnant Australian Indigenous women and their antenatal care providers. Cross-sectional surveys of staff and women in antenatal services providing care for Indigenous women in the Northern Territory and New South Wales, Australia. Respondents were asked to indicate the extent to which each of a list of possible strategies would be helpful in supporting pregnant Indigenous women to quit smoking. Current smokers (n = 121) were less positive about the potential effectiveness of most of the 12 strategies than the providers (n = 127). For example, family support was considered helpful by 64 % of smokers and 91 % of providers; between 56 and 62 % of smokers considered advice and support from midwives, doctors or Aboriginal Health Workers likely to be helpful, compared to 85–90 % of providers. Rewards for quitting were considered helpful by 63 % of smokers and 56 % of providers, with smokers rating them more highly and providers rating them lower, than most other strategies. Quitline was least popular for both. This study is the first to explore views of pregnant Australian Indigenous women and their antenatal care providers on strategies to support smoking cessation. It has identified strategies which are acceptable to both providers and Indigenous women, and therefore have potential for implementation in routine care. Further research to explore their feasibility in real world settings, uptake by pregnant women and actual impact on smoking outcomes is urgently needed given the high prevalence of smoking among pregnant Indigenous women.

## Introduction

Tobacco smoking among pregnant Aboriginal and Torres Strait Islander women remains three times as common as among non-Indigenous Australian pregnant women, with approximately 50 % of women smoking during pregnancy [1]. Addressing this disparity is a priority for reducing the gap in health outcomes between Indigenous and non-Indigenous Australians. Disparities in smoking rates between Indigenous and non-Indigenous pregnant women are also marked in the United States, Canada and New Zealand [2–4]. While interventions to reduce antenatal smoking are known to be effective in non-Indigenous populations [5], to date effective interventions for pregnant Indigenous women have not been identified [6–8].

Previous reviews of interventions for smoking cessation in Indigenous peoples have concluded that approaches that specifically target Indigenous populations can be successful [9, 10], and that interventions targeting individuals, such as counselling and nicotine replacement therapy (NRT), which are known to be effective in other populations, are likely to be effective for Indigenous people [11]. However, these reviews did not include trials with pregnant Indigenous women. A review of smoking cessation interventions specifically for pregnant Indigenous women identified only two relevant trials, neither of which increased cessation, highlighting the need for further research to identify effective strategies [8]. In addition to considering approaches found to work in other pregnant population groups, a useful starting point for developing interventions is an exploration of the views of pregnant Indigenous women, and the staff providing their antenatal care.

## Aims

To assess support for a range of potential smoking cessation program strategies among pregnant Indigenous women who currently smoke tobacco, pregnant ex-smokers, and their antenatal care providers.

## Methods

Cross-sectional surveys with antenatal care providers and pregnant Indigenous women were undertaken in the Northern Territory (NT) and New South Wales (NSW). The project was guided by a community reference group (CRG) to ensure cultural security. The CRG was composed of Aboriginal women from the community (some of whom were pregnant), Aboriginal Health Workers (AHWs) and Community Midwives. Ethical approval for the research was provided by the Human Research Ethics Committees of the University of Newcastle, the NT Department of Human Services and Menzies School of Health Research, Hunter New England Health Service and the Aboriginal Health & Medical Research Council of NSW.

### Recruitment

The detailed methodology for both surveys is described elsewhere [12, 13]. A brief summary follows.

#### Staff Survey

Briefly, staff providing antenatal care in remote medical services in the NT and through the Aboriginal Maternal and Infant Health Service (AMIHS) in NSW were eligible and were identified by their relevant health departments and services. All staff worked in community based services. Between September 2008 and July 2009, eligible staff were sent invitation letters, information sheets and self-completion questionnaires. They were asked to complete the anonymous questionnaires and return them in pre-paid envelopes. Reminder letters with additional copies of the documents were sent twice—3 weeks after the initial invitation and again 1 month later. Return of the questionnaire was considered to imply consent.

#### Women’s Survey

Women were recruited by the AMIHS teams from July to December 2009, and from the maternity outpatient clinic of a major hospital from July to September 2010 and April to June 2011. Women were eligible if pregnant and if they or their partner were Indigenous. They were excluded if aged less than 16; being treated for mental illness; or unable to provide informed consent. Consecutive eligible women were invited to participate by the midwife, AHW or a female Aboriginal research assistant, who explained the study and provided women with information sheets. Written consent was obtained. Recruiting staff offered assistance to complete the questionnaire if required. Staff were asked to invite all eligible women to participate and to complete a recruitment log to track participation rates.

### Questionnaire Development and Contents

Draft questionnaires were critically reviewed by the CRG and colleagues experienced in Indigenous health research and smoking cessation, to assess content validity, reduce redundancy and refine the wording to ensure cultural appropriateness. Minor revisions were made prior to pilot-testing with 12 antenatal service providers, and 15 pregnant Indigenous women, in NSW and Western Australia. Further minor modifications were made in consultation with the CRG.

The final questionnaires had Flesch-Kincaid reading levels of grade 9 (staff) and grade 6 (women) and both took 15–20 min to complete. The questionnaires for staff and women differed with regards to some content, but of relevance to this paper, both included a question on strategies. For staff, the wording was “Please indicate how useful you think each of the following would be in helping pregnant women quit smoking”. They were then presented with a list of 12 possible strategies, and asked to indicate if they considered them to be ‘very helpful’, ‘somewhat helpful’, ‘maybe helpful’, ‘not helpful’ or ‘harmful’. The women were asked “How useful do you think each of the following would be in helping pregnant women to quit smoking”, with the same list of strategies and response options. Additionally, both the staff and women’s questionnaires included a question on smoking status—current daily smoker, current occasional smoker, ex-smoker or never smoked. The women’s questionnaire also asked the usual number of cigarettes smoked each day; and their age, education, and parity.

### Statistical Analysis

Responses to the question on smoking status were categorised into current smokers (current daily or occasional smokers), ex-smokers or never smokers. Responses to the questions on the helpfulness of the strategies were dichotomised into ‘very or somewhat helpful’ or ‘other’. For the women’s survey, only responses from smokers and ex-smokers were included in the analysis.

Summary statistics of respondent characteristics were obtained. For the women, mean age and number of cigarettes smoked were calculated. Years of school, and parity were categorised, and the number and percentage in each category reported. For the staff, the number and percentage for each profession was calculated.

The proportion of each group (women who were current smokers, ex-smokers and service providers) who considered each strategy ‘very or somewhat helpful’ was calculated and 95 % confidence intervals generated. We also assessed the proportions in each group indicating that each strategy ‘maybe helpful’, was ‘not helpful’ or was ‘harmful’.

## Results

### Description of the Sample

In total, 264 women responded to the survey, of whom 121 were current smokers and 55 were ex-smokers and included in this analysis. The response rate could not be calculated as not all teams returned recruitment logs, but among the teams which did, the response rate was 88 %. The majority of smokers (85, 70 %) reported smoking every day with the remaining 36 (30 %) smoking occasionally. The smokers reported an average of 10 cigarettes per day. Other characteristics of the current smokers and ex-smokers are presented in Table 1.

**Table 1:** Characteristics of women who were current smokers (n = 121) and ex-smokers (n = 55)

	Current smokers n = 121	Ex-smokers n = 55
Age: mean (standard deviation)	24.9 (5.69)	24.4 (6.02)
Completed year 12 at school: n (%)	14 (12)	10 (18)
Post-secondary education: n (%)	40 (33)	32 (58)
Primiparous: n (%)	29 (24)	21 (38)

127 of 184 (69 %) eligible service providers responded, of whom 30 (24 %) were AHWs, 89 (70 %) were nurses or midwives, and eight (5 %) were doctors. Nineteen (15 %) reported being current smokers [10 AHWs (33 %) and nine midwives (10 %)].

### Perceived Helpfulness of Suggested Strategies

The numbers of participants indicating that they thought each strategy would be very or somewhat helpful for pregnant women in quitting smoking are shown in Table 2 and are presented in order of the proportion of current smokers indicating they thought the strategy would be helpful. Overall, a greater proportion of service providers were likely to consider each of the strategies helpful than the current smokers, with the ex-smokers generally between the providers and the current smokers. Four of the six strategies rated most highly by smokers (family support, advice and support from the midwife, doctor or AHW) were also in the top five supported strategies for ex-smokers and the top four for providers. Interestingly, rewards were the most popular strategy among ex-smokers (83 %) and the 2nd most popular with current smokers (63 %) but equal 10th among providers (56 %). Community activities were less supported by ex-smokers (51 %) than by either current smokers (59 %) or providers (74 %). Access to Quitline was supported by less than 50 % of respondents in all three groups. For each strategy, respondents who did not consider it likely to be helpful were split fairly evenly between ‘maybe helpful’ and ‘not helpful’. The only strategies considered harmful by more than one person in any group were free NRT which was considered harmful by eight providers, five current smokers and one ex-smoker; and rewards for quitting which were considered harmful by six providers, one current smoker and one ex-smoker (not shown in table).

**Table 2:** Proportion of respondents considering each strategy very or somewhat helpful among antenatal service providers, pregnant women who smoke and pregnant ex-smokers

Strategy^a,b^	Very or somewhat helpful					
	Women					
	Current smokers N = 121		Ex-smokers N = 55		Service providers N = 127	
	n	% (95 % CI)	n	% (95 % CI)	n	% (95 % CI)
Support for the whole family to help others quit	74	64 (54, 73)	40	74 (60, 85)	116	91 (85, 96)
Rewards for women who stop smoking with vouchers to get things for the mother or baby	74	63 (54, 72)	43	83 (70, 92)	70	56 (46, 64)
Advice and support from the midwife	74	62 (53, 71)	39	74 (60, 85)	108	86 (78, 91)
Advice and support from the doctor	72	61 (52, 70)	41	76 (62, 87)	108	85 (78, 91)
Community activities about quitting	68	59 (49, 68)	27	51 (37, 65)	93	74 (66, 82)
Advice and support from the AHW	66	56 (47, 66)	41	76 (62, 87)	114	90 (83, 94)
Free nicotine replacement therapy	66	56 (47, 66)	33	62 (48, 75)	92	74 (66, 82)
Peer support groups	60	53 (45, 62)	40	74 (60, 85)	102	81 (73, 87)
Brochures: harms of smoking and advice on quitting	61	52 (43, 61)	27	50 (36, 64)	71	56 (47, 65)
Stress management programs	57	49 (39, 58)	38	70 (56, 82)	92	73 (64, 81)
Support person	55	47 (38, 57)	37	69 (54, 80)	79	63 (54, 71)
Access to a Quitline	54	46 (37, 56)	26	49 (35, 63)	61	49 (40, 58)

^a^Ordered by proportion of current smokers perceiving strategies to be very or somewhat helpful

^b^0–5 missing responses for each variable

## Discussion

This paper is the first we are aware of that explores the degree to which pregnant Indigenous women and antenatal care providers consider particular strategies helpful for antenatal smoking cessation. In general, current smokers were least supportive of most strategies, and providers were most supportive. The majority of strategies were supported by over half the participants in each group. The reasons for the lower support among current smokers than among ex-smokers and providers on most strategies is not known, but may reflect their personal struggles with quitting and recognition of the difficulty of quitting or a general sense of hopelessness regarding the prospects of success. While these results reflect the opinions of respondents, not the actual efficacy of strategies, establishing acceptability is a useful starting point for developing intervention trials.

### Rewards for Smoking Cessation

A similar proportion of current smokers and providers considered rewards likely to be helpful (63.3 and 55.6 % respectively) but a higher proportion of ex-smokers indicated they thought rewards would be helpful (83 %), with rewards the most popular strategy in this group, 2nd most popular among current smokers and equal 10th among providers.

The Cochrane review of antenatal smoking cessation interventions identified provision of incentives, or rewards, as the most effective intervention, with incentives reducing smoking by 24 % compared to 6 % for all interventions combined [5]. Incentives are considered most effective for simple, time-limited behaviours such as completing immunisation, but may be less effective where the required behaviour change is complex [14]. For maintaining complex behaviour change, financial incentives may be a useful addition to multi-faceted programs that address the complex individual, social and economic factors affecting behaviour [14].

Incentives for antenatal smoking cessation are already used in some parts of the British NHS [15, 16], yet their use for health behaviour change remains controversial [15]. In a survey of pregnant Australian women, the majority did not support paying pregnant smokers to quit, but smokers were more likely to do so [17]. A qualitative study with social service staff and clients found that clients were supportive of rewards for quitting while staff were less so, and expressed concerns about the feasibility of implementation [18]. Our results add to this body of work identifying significantly greater support for rewards among ex-smokers than among providers. Given the apparent efficacy of incentives in antenatal smoking cessation, further research is required to explore the reasons for the low support among providers relative to their support for other strategies.

### Involving Family

The strategy rated highest by both current smokers and providers was “support for the whole family to help others quit”. Smokers who are supported by their partners are more likely to succeed, but a recent systematic review of interventions aimed at enhancing partner support to improve smoking cessation found little evidence for effective interventions [19]. Family based interventions have been recommended for Indigenous Australians because of the importance of family in influencing smoking behaviour [20, 21]. The endorsement by women and service providers in our study provides additional evidence for their acceptability and further support for their inclusion in future trials to assess their efficacy.

### Health Professionals

Advice and support from the range of health professionals were each rated reasonably highly by all groups. Good evidence exists for efficacy of advice from doctors and nurses [22, 23], however midwives, including midwives caring for Indigenous women, have expressed reluctance to address smoking, concerned that they may damage their relationship with their clients [12, 24]. Similar concerns have been expressed by AHWs, with the additional concern that AHW smoking may impede providing advice [25]. However, over half the women in our study indicated that support from each of the health professionals was likely to be helpful, suggesting this approach is acceptable, perceived to be effective and may be a fruitful approach.

### Other Strategies

Community activities were rated fifth and sixth by current smokers and providers respectively but 10th by ex-smokers. The reasons for the lower support among ex-smokers are not known. Previous studies have emphasised the preference of Indigenous Australians for programs to be community-based [21]. Although community interventions increase knowledge of risks, change attitudes to smoking and increase quit attempts, they have not been shown to reduce the prevalence of smoking [26, 27].

Other activities considered helpful by at least half of each group included free NRT, support groups and brochures. NRT is efficacious in non-pregnant populations, but evidence for its effectiveness in pregnancy is inconclusive [28]. Pregnant Indigenous women have previously been found to have relatively low levels of nicotine dependency [29], which may contribute to the lower rating for NRT in our study. Current guidelines state that NRT should be considered if a pregnant woman is otherwise unable to quit [30], and it would therefore be reasonable to include free NRT as a component of future cessation trials. In non-pregnant populations, group programs are more effective than self-help and other low intensity interventions, but the limited research in this area has not provided an adequate evidence base to determine whether they are more effective than intensive individual counselling, or whether they provide additional benefit as an adjunct to individual support [31]. Although generally supported by respondents in each group, the logistic challenges of running groups, particularly in rural areas, would need to be overcome if they were to be included in future smoking cessation trials. Low intensity interventions, including providing verbal or written advice, demonstrated a small benefit in the Cochrane review on antenatal smoking cessation [5]. While unlikely to have a large impact, culturally appropriate brochures and other resources may be a useful prop to use when discussing smoking cessation.

Interestingly, less than half the current smokers thought that stress management programs would be helpful. Research on smoking among Indigenous Australians has emphasised stress as an impediment to cessation [21, 32]. Although stress contributes to pregnant women failing to quit, and stress management techniques are included in some cessation programs, the evidence on their benefit is inconclusive [33].

### Limitations

A number of limitations need to be considered in interpreting the results from this study. The response rate was higher among the women than the service providers. The reasons for this difference are unknown, but it may be due to differences in recruitment, with providers recruited by letter, and women recruited through a personal approach. Secondly, the sample is fairly small, despite the reasonably good response rates. However, Indigenous women are a small proportion of the population, and engaging them in research can be challenging. One of the strengths of this study is that it includes women from across two different states, and they are representative of pregnant Indigenous women nationally with regard to age and parity [34]. Thirdly, a delay between the providers’ and the women’s surveys may have impacted on the results. However, we are unaware of any specific programs or initiatives which occurred between the two surveys that could be considered to impact on the findings. A fourth limitation is that the data are drawn from cross-sectional surveys, with no opportunity to explain the proposed strategies in more detail, nor to explore the reasons for support or opposition to the strategies. More importantly, the apparent support may not translate into implementation or uptake, nor into actual changes in smoking behaviour. Further intervention research is required to explore the feasibility of implementing these strategies in real world settings, their uptake by pregnant women and their actual impact on smoking rates and health outcomes.

## Conclusions

Exploring the views of stakeholders involved in antenatal smoking cessation—the providers and the pregnant women, has identified the strategies which are most acceptable, and thus the ones most likely to be implemented if introduced in routine care. These strategies, if known to be effective in other pregnant populations, should be included in interventions and tested in trials to assess their real world uptake and their impact on smoking behaviours and health outcomes. Given the apparent efficacy of rewards in other population groups, further research is required to assess their efficacy among pregnant Indigenous women and to identify reasons for their lower support by providers.
